# Sleep quality and adherence to medical therapy among hemodialysis patients with depression: a cross-sectional study from a developing country

**DOI:** 10.1192/bjo.2021.164

**Published:** 2021-06-18

**Authors:** Syed Muhammad Jawad Zaidi, Mehwish Kaneez, Javeria Awan, Hamza Waqar Bhatti, Muhammad Hamza, Mishal Fatima, Muhammad Zubair Satti

**Affiliations:** Rawalpindi Medical University

## Abstract

**Aims:**

Depression is a fairly common finding among end-stage renal disease (ESRD) patients on hemodialysis and is an independent risk factor for morbidity and mortality. The psychiatric manifestations of the disease may affect their compliance to medications and alter sleep quality that is often overlooked by nephrologists. This translates into poor quality of life and poorer disease prognosis. Thus, Our study aims to assess the prevalence of depression and its association with compliance to medical therapy and sleep quality among ESRD patients on hemodialysis.

**Method:**

In this cross-sectional study, a total of 288 hemodialysis patients with a confirmed diagnosis of ESRD were evaluated for depression using Patient Health Questionnaire-9 (PHQ-9) scale. Only the patients with moderate to severe depressive symptoms on PHQ-9 were further evaluated for sleep quality and compliance to medications using the Pittsburgh Sleep Quality Index (PSQI) and Drug Attitude Inventory-10 (DAI-10) respectively. The characteristics of ESRD patients with depression were also assessed. Median PHQ-9, DAI-10, and PSQI scores were calculated and the correlation between study variables was assessed using spearman's correlation.

**Result:**

Of the 288 included participants, 188 (65.27%) had depression as evaluated via PHQ-9. Of these 188 patients, 114 were males while 74 were females. A total of 113 (60.01%) of the depressed patients had poor compliance with medication while 137 (72.87%) patients had poor sleep quality. Higher PHQ-9 scores were positively correlated with disease duration, dialysis years, and time between diagnosis and therapy (r = 0.41, 0.39, and 0.43 respectively) and negatively with marital and employment status (r = −0.32 and −0.49 respectively). Spearman's correlation matrix showed that PHQ-9 scores were negatively correlated with DAI-10 but positively correlated with PSQI scores.

**Conclusion:**

The study indicates a high prevalence of depression among ESRD patients on hemodialysis. Poor sleep quality and non-adherence to medications are extremely common among ESRD patients with depression. These psychiatric components must be considered to optimize medical treatment and improve the quality of life in this subset of patients.

